# Prevalence of high‐risk plasma p‐tau217 levels and 5‐year transition of risk status in 70‐year‐olds

**DOI:** 10.1002/alz.71545

**Published:** 2026-06-07

**Authors:** Anna Dittrich, Burak Arslan, Tobias Skillbäck, Lina Rydén, Hlin Kvartsberg, Johan Gobom, Kaj Blennow, Henrik Zetterberg, Silke Kern, Ingmar Skoog

**Affiliations:** ^1^ Neuropsychiatric Epidemiology Unit Sahlgrenska Academy University of Gothenburg Mölndal Sweden; ^2^ Department of Neuropsychiatry Sahlgrenska University Hospital Gothenburg, Region Västra Götaland Sweden; ^3^ Department of Psychiatry and Neurochemistry, Institute of Neuroscience and Physiology the Sahlgrenska Academy at the University of Gothenburg Gothenburg Sweden; ^4^ Clinical Dementia Research, Sahlgrenska Academy University of Gothenburg Gothenburg Sweden; ^5^ Clinical Neurochemistry Laboratory Sahlgrenska University Hospital Mölndal Sweden; ^6^ Department of Pathology and Laboratory Medicine University of Wisconsin School of Medicine and Public Health Madison Wisconsin USA; ^7^ Centre for Brain Research Indian Institute of Science Bangalore Karnataka India; ^8^ Department of Neurodegenerative Disease UCL Institute of Neurology London UK; ^9^ UK Dementia Research Institute at UCL London UK; ^10^ Hong Kong Center for Neurodegenerative Diseases Hong Kong New Territories China; ^11^ Wisconsin Alzheimer's Disease Research Center, University of Wisconsin School of Medicine and Public Health University of Wisconsin‐Madison Madison Wisconsin USA

**Keywords:** Alzheimer's disease, biomarkers, cohort study, community‐based, dementia, longitudinal, plasma p‐tau217

## Abstract

**INTRODUCTION:**

High‐risk plasma p‐tau217 levels predict amyloid‐β pathology in Alzheimer's disease, but little is known about the prevalence and temporal dynamics in the general population.

**METHODS:**

Participants from the Gothenburg H70 Birth Cohort Study in Sweden were examined (*n* = 1157) and re‐examined after 5–7 years (*n* = 771). Prevalence and 5–7 year transition of high‐risk plasma p‐tau217 status was determined among community‐dwelling 70‐year‐olds.

**RESULTS:**

High‐risk plasma p‐tau217 prevalence was 3.6% at age 70 and 7.0% at age 75–77 years. Eighty‐nine percent remained low‐risk and 4% converted to high‐risk at follow‐up. Prevalence of dementia at follow‐up was 1.7% if remaining in the low‐risk group and 21.4% if transitioning to the high‐risk group. Prevalence of dementia among participants staying in the high‐risk group was 16.7% at follow‐up.

**DISCUSSION:**

Individuals with low‐risk plasma p‐tau217 were unlikely to transition over 5–7 years. However, transitioning to a higher risk‐category was associated with a higher prevalence of cognitive disability.

## Highlights

- The prevalence of high‐risk plasma p‐tau217 levels was 3.6% at age 70.
- The prevalence of high‐risk plasma p‐tau217 levels was 7.0% at age 75–77.
- Plasma p‐tau217 showed a high stability over a 5–7 years in low‐risk individuals.
- Cognitive disability was higher among those transitioning to a high risk‐category.
- The high‐risk group had a 16.7% prevalence of dementia at follow‐up.

## 1 BACKGROUND

With the recent advancements in disease‐modifying treatments for Alzheimer's disease (AD),^1^ availability of easily accessible biomarkers for disease detection is rapidly increasing.^2^ Blood‐based tests have the benefit of being easily implemented early, already in a primary care setting, and can provide an excellent basis for early diagnostics.^3^ Plasma p‐tau217 is currently one of the best performing plasma biomarkers in AD^4^ and has recently shown to accurately predict amyloid‐β (Aβ) pathology in a clinical setting^5^ utilizing a two‐cutoff approach.

In clinical samples, plasma p‐tau217 has been found to have a positive predictive value above 95% in patients with suspected AD, and rule out AD in patients with symptoms of other dementias with a negative predictive value between 90% and 99%.^6^ High plasma p‐tau217 has also been shown to predict cognitive decline in cognitively unimpaired patients with preclinical AD from memory clinics.^7^ In a study of cognitively healthy participants, high plasma p‐tau217 was associated with an increased risk for Aβ pathology over time.^8^ In studies recruiting from both the community and clinical settings, plasma p‐tau217 has been shown to be one of the best blood‐based analytes to predict high amyloid deposition in the brain.^9^ In the Mayo Clinic Study of Aging plasma p‐tau217 has previously been demonstrated to correlate with amyloid‐ and tau‐PET examinations.^10^ Strictly community‐based studies are currently limited, but the predictive capacity of amyloid PET in relation to plasma p‐tau217 has since been replicated in another community‐based cohort.^11^

While it is well established that the prevalence of Aβ pathology increases over the life span,^12^ little is known regarding the temporal dynamics of plasma p‐tau217 among individuals in a general population. Here we examine prevalence of high‐risk plasma p‐tau217 levels and the five‐year transition rate in p‐tau217 status among community‐dwelling 70‐year olds in Gothenburg, Sweden and assess the cognitive status for each risk group.

**RESEARCH IN CONTEXT**

1. **Systematic review**: The authors searched PubMed for articles on plasma‐p‐tau217 in a community‐based setting and found evidence from diverse populations population based studies across large parts of the world on a capacity to predict cognitive decline, as well as predict amyloid deposition determined through positron emission tomography (PET)‐camera. However, studies reporting age‐specific prevalence of pathological levels, and repeated measurements of plasma‐p‐tau217 with implications of the temporal dynamics were missing.
2. **Interpretation**: Our observations from the longitudinal H70 Birth Cohort study in Gothenburg, Sweden presents the first data on prevalence of high‐risk plasma p‐tau217 levels in 70 and 75 year–old community‐dwellers. We also demonstrate the temporal dynamics to transition into high‐risk status over 5–7 years with 21.4% prevalence of dementia among participants transitioning to the high‐risk group over the follow‐up time.
3. **Future directions**: We conclude that plasma‐p‐tau217 levels remain stable over 5–7 in the vast majority of low‐risk individuals, and that transition to the high‐risk category is paralleled with a much higher frequency of dementia. This should be considered when clinically determining the frequency of re‐testing low‐risk individuals with plasma‐p‐tau217, and highlights the importance of further cognitive assessment of individuals who transition in plasma‐p‐tau217 after repeated testing.

## 2 METHODS

### 2.1 Design

The H70 Birth Cohort Study 1944 is a 5‐ to 7‐year longitudinal, population‐based study of 70‐year‐olds living in Gothenburg, Sweden. The primary outcome for this study was to determine the rate of transition between individuals stratified into low‐, intermediate‐, and high‐risk of Aβ pathology measured by plasma p‐tau217 between baseline and follow‐up.

### 2.2 Participants

**FIGURE 1:**
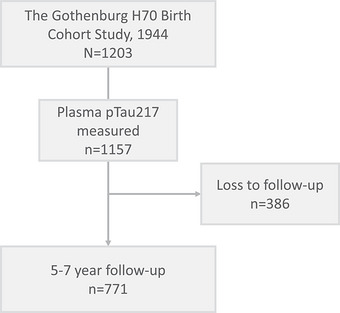
Flow‐chart of the study.

In 2014‐2016, a representative sample of 70‐year‐olds born on certain dates during 1944 and living in Gothenburg (*N* = 1203, response rate 72%, mean age 70.5 years, 559 men and 644 women, 96.5% born in Europe) (Figure 1) were examined as described previously.^13^ All individuals born on the prespecified birth dates and living in Gothenburg were invited to ascertain a sample fully representative of the general community. In 2019‐2022, a follow‐up study on 75‐year‐olds was conducted, re‐examining the initial participants (age 75 examination). This study was interrupted due to the pandemic (influence detailed under results). Finally, 902 individuals were examined (response rate 61.8%, mean age 76.6 years, men 423 and women 479).

Participants were stratified into three risk categories for prevalent Aβ pathology based on plasma p‐tau217 cutoffs established in our laboratory across several cohorts, as previously described (< 0.22 and > 0.34 pg/mL, with values above > 0.34 pg/mL indicating 95% risk of Aβ pathology)^5^. At baseline, 1157 participants had plasma p‐tau217 measured. Among those re‐examined at the age of 75, 791 had plasma p‐tau217 measured, resulting in 771 having plasma p‐tau217 measured at both baseline and at follow‐up, constituting the samples for this study (Figure 1). The study determines prevalence of high‐risk plasma p‐tau217 levels among all participants with valid measurements at baseline, and conducts longitudinal analyses of those with valid measurements at both baseline and follow‐up.

### 2.3 Examinations

Participants were examined with health interviews, cognitive testing, blood sampling, and physical examinations at baseline and follow‐up at the Neuropsychiatric Clinic at Sahlgrenska University Hospital in Gothenburg, Sweden. Anthropometric variables included weight, height, and body mass index (BMI). All participants without contraindications were invited to a lumbar puncture (LP). Participants accepting LP (238 out of 771 participants) did not differ from the full sample in Clinical Dementia Rating (CDR) scale score or apolipoprotein E (*APOE)* genotype, but had a lower prevalence of stroke and use of anticoagulants due to contraindication for LP.^14^

CDR was assessed by specifically trained research nurses^13^ and dementia was diagnosed according to the Diagnostic and Statistical Manual of Mental Disorders, 3rd edition, revised criteria. Mild cognitive impairment (MCI) was defined as CDR = 0.5, stroke if this was reported by the participant, a close relative, or reported in the Swedish National patient register. A self‐reported history of diabetes defined diabetes and eGFR below 60 mL/min/1.73 m^2^ defined chronic kidney disease (CKD).^15^

### 2.4 Biomarker analyses

Fasting glucose and creatinine were analysed from venous blood at the Sahlgrenska Clinical Chemistry laboratory and estimated glomerular filtration rate (eGFR) calculated.^16^ *APOE* genotyping was done using the KASPar polymerase chain reaction (PCR) single‐nucleoride polymorphism (SNP) genotyping system (LGC Genomics, Hoddesdon, Herts, UK).

LP was done within 2 months of an magnetic resonance imaging (MRI) examination^13^. In cerebrospinal fluid (CSF), t‐tau and p‐tau181 were analyzed using enzyme‐linked immunosorbent assays (ELISA) (INNOTEST htau Ag and PHOSPHO_TAU [181P], Fujirebio [formerly Innogenetics], Ghent, Belgium),^17^, ^18^ Aβ42 using the INNOTEST® Aβ1‐42 ELISA.^19^ Aβ42/40 using the V‐PLEX Aβ Peptide Panel 1 (6E10) Kit (MesoScale Discovery, Rockville, MD)^20^ and CSF neurofilament light chain (NfL) using an ELISA developed at the Mölndal Clinical Neurochemistry Laboratory.^21^ Analytical runs passed quality control criteria as previously described.^22^ Aβ pathology status was defined as Aβ42 levels below 530 pg/mL.^23^

### 2.5 Lumipulse plasma p‐tau217

Plasma p‐tau217 concentrations were quantitatively measured using Lumipulse G p‐tau217 cartridges (Lot: D4C5023) and substrate (Lot: 5050), based on chemiluminescent enzyme immunoassay (CLEIA) technology on the fully automated LUMIPULSE® G1200 platform (Fujirebio, Japan). Prior to analysis, the assay was calibrated (Calibrator Lot: D5C5098), and runs were validated using commercial quality controls (QC Lot: D6C5096). Repeatability (%CV_r_) and intermediate precision (%CV_Rw_) were 5.4% and 5.7% for low QC (mean: 0.5 pg/mL), and 2.5% and 6.0% for high QC (mean: 3.8 pg/mL), respectively.

### 2.6 Statistics

Data are presented as mean (SD) for continuous variables and n (%) for categorical variables. Comparisons between two groups were done with Student's t‐test, paired comparisons with paired t‐test, and comparisons between three groups with analysis of variance (ANOVA). Differences in categorical variables were tested with chi‐squared test or Fishers exact test. A *p*‐value < 0.05 defined statistical significance.

## 3 RESULTS

### 3.1 Loss to follow‐up

Out of the 1157 participants with plasma p‐tau217 measured at baseline, 386 were lost to follow‐up (characteristics in Table S1). There was a progressively higher proportion of participants lost to follow‐up with higher plasma p‐tau217 risk classification. In the low‐risk group 338 (32%) participants were lost to follow‐up, in the intermediate‐risk group 20 (42%), and in the high‐risk group 28 (67%). Reasons for loss to follow‐up (declining re‐examination, death etc) did not differ between risk groups. CKD and *APOE ε4‐*carriership were more common in the high‐risk group among participants lost to follow‐up.

The follow‐up period lasted over the covid‐19 pandemic and around half of the participants had their re‐examination before the pandemic (*n* = 370, 53.5% females) and half after the start of the pandemic (*n* = 401, 51.1% females). There was a brief pause in examinations between March in 2020 and September 2020 as well as between December 2020 and March 2021. There was no difference in plasma p‐tau217 levels between participants re‐examined before or after the start of the pandemic.

### 3.2 Prevalence of high‐risk plasma p‐tau217 among 70‐ and 75‐year‐olds

The prevalence of high‐risk plasma p‐tau217 levels at age 70–72 years was 3.6% (females 3.9%, males 3.3%). Low‐risk levels of plasma p‐tau217 were found in 92.2% of all participants, indicating a low‐risk of amyloid pathology by plasma p‐tau217 levels (Table 1). This prevalence was similar between females and males (92.3% and 92.1% respectively).

**TABLE 1:** Prevalence of plasma p‐tau217 risk‐categorization at age 70 and 75 (5‐ to 7‐year follow‐up).

Parameter	Low risk	Intermediate risk	High risk
**Age 70 years (*n* = 1157)**			
All	1067 (92.2)	48 (4.1)	42 (3.6)
Females	565 (92.3)	23 (3.8)	24 (3.9)
Males	502 (92.1)	25 (4.6)	18 (3.3)
**Age 75–77 years (*n* = 771)**			
All	653 (84.7)	64 (8.3)	54 (7.0)
Females	345 (85.6)	36 (8.9)	22 (5.5)
Males	308 (83.7)	28 (7.6)	32 (8.7)

*Note*: Data presented as n (%).

The prevalence of high‐risk plasma p‐tau217 levels at age 75–77 years was almost twice as high (7.0%) with higher prevalence found in both sexes (5.5% in females and 8.7% in males) compared to the prevalence at age 70–72. The prevalence with low‐risk levels was lower overall (all 84.7%, females 85.6%, males 83.7%).

### 3.3 Characteristics of participants stratified by plasma p‐tau217 at baseline and follow‐up

Among the 771 participants with plasma p‐tau217 measured at baseline and follow‐up, there were no differences in cognitive function or somatic comorbidities between the three risk groups at study baseline (Table 2). For biomarkers, the low‐risk group of plasma p‐tau217, *APOE4* carriership and CSF levels of tau, p‐tau181, and NfL were lowest, and Aβ42/40 levels were highest, with statistical differences only to the intermediate risk group (Table 2).

**TABLE 2:** Characteristics of participants in the Gothenburg H70 with plasma pTau217 measured at both baseline and follow‐up (*n* = 771).

	Baseline (age 70–72 years)				Follow‐up (age 75–77 years)			
Parameter	Low risk	Intermediate risk	High risk	*p‐*Value	Low risk	Intermediate risk	High risk	*p‐*Value
n (%)	729 (94.6)	28 (3.6)	14 (1.8)		653 (84.7)	64 (8.3)	54 (7.0)	
Age, years	70.5 (0.3)	70.5 (0.3)	70.7 (0.4)	0.052	76.3 (0.6)	76.3 (0.7)	76.4 (0.6)	0.225
Sex, female	386 (53)	12 (43)	5 (36)	0.264	345 (53)	36 (56)	22 (41)	0.186
MMSE, score	29.1 (1.3)	28.8 (1.5)	29.3 (1.3)	0.502	28.8 (1.5)	28.1 (2.1)^*^	27.7 (2.9)^*^	**<0.001**
CDR = 0	607 (83)	22 (79)	11 (79)	0.733	515 (79)	43 (67)	27 (50)^*^	**<0.001**
CDR = 0.5	116 (16)	6 (21)	3 (21)	0.641	134 (21)	21 (33)	21 (39)^*^	**0.001**
CDR = 1‐3	4 (1)	0 (0)	0 (0)	1.000^a^	4 (1)	0 (0)	6 (11)^*^	**<0.001** ^a^
BMI, kg/m2	25.8 (4.2)	24.9 (5.8)	27 (3.9)	0.353	25.8 (4.3)	24.9 (4.5)	24.8 (4.4)	0.144
Dementia	6 (1)	0 (0)	0 (0)	1.000^a^	11 (2)	6 (9)^−^	10 (19)^*^	**<0.001**
Previous stroke	40 (5)	3 (11)	0 (0)	0.348^a^	45 (7)	7 (11)	5 (9)	0.435
Diabetes	64 (9)	4 (14)	2 (14)	0.308^a^	87 (13)	7 (11)	10 (19)	0.465
CKD	59 (8)	3 (11)	1 (7)	0.725^a^	81 (13)	17 (27)^*^	11 (20)	**0.004**
*APOE*ε*4‐*carrier	222 (30)	17 (61)^*^	5 (36)	**0.003**	189 (29)	34 (53)^*^	21 (39)	**<0.001**
CSF‐substudy (H70, n = 238)								
N	226	7	5					
Aβ42	557.1 (181)	400 (208.9)	484.2 (289.6)	0.062				
Aβ42/40	0.9 (0.2)	0.6 (0.2)^*^	0.8 (0.3)	**<0.001**				
Tau, pg/mL	314 (117.6)	524.4 (178.5)^*^	338.2 (60.8)	**<0.001**				
pTau, pg/mL	47.4 (15.4)	72 (23.2)^*^	60.8 (25.9)	**<0.001**				
Neurofilament Light Chain, pg/mL	881.9 (804.4)	804.3 (220)	3041 (5192.8)^*^	**<0.001**				
Q_Alb_	6.5 (2.5)	6.1 (1.6)	5.9 (0.7)	0.778				
Aβ‐pathology	101 (45)	6 (86)	2 (40)	0.096				

*Note*: Continuous variables are presented as mean (SD) and categorical variables as n (%). Continuous variables were compared with one‐way ANOVA and categorical variables with chi‐squared test or Fishers exact test. Participants are stratified according to cutoffs for plasma pTau217 at each respective timepoint.

^a^ If low frequency of events.

Abbreviation: Aβ, amyloid‐β; ANOVA, analysis of variance; APOE, apolipoprotein E; BMI, body mass index; CDR, Clinical Dementia Rating; CKD, chronic kidney disease; CSF, cerebrospinal fluid; MMSE, Mini‐Mental State Examination; NfL, neurofilament light chain; Q_Alb_, albumin quotient.

* Difference compared to low‐risk group, with Bonferroni‐adjusted z‐test for categorical variables or Dunett's post hoc test for continuous variables.

When re‐examined 5‐7 years later and plasma p‐tau217 was measured again, higher plasma p‐tau217 risk group at follow‐up was associated with worse cognitive function measured as all stages of CDR as well as manifest dementia (Table 2). Consistent with the baseline results, there was an association with *APOE4* carriership, but at follow‐up, also with a medical history of CKD. There was no statistically significant increase in the proportion of MCI (CDR = 0.5) or dementia over 5–7 years of follow‐up in the small group at high risk (*n* = 14, Table S2). However, there was a slight decrease in MMSE score (mean ± SD, 29,3 ± 1,3 to 28,4 ± 2,3, *p *= 0.031).

The increase in plasma p‐tau217 concentrations was higher among individuals who increased in CDR from 0 at baseline to a higher value at follow‐up (0.02 [0.00–0.06] pg/mL for those remaining in CDR = 0 and 0.03 [0.01‐0.08] pg/mL for those increasing, median [interquartile range {IQR}], *p*‐value = 0.01). Similarly, increases in plasma p‐tau217 were higher in individuals who increased from CDR 0.5 at baseline to a higher CDR at follow‐up (0.02 [0.00–0.04] pg/mL and 0.25 [0.28–0.45] pg/mL respectively, median [IQR], *p*‐value = 0.001). On individual level, there was a substantial overlap between groups, and the discriminative value for using the change in plasma p‐tau217 over 5 years to estimate progression in CDR on individual level was low.

### 3.4 Transitions between risk groups over time

**FIGURE 2:**
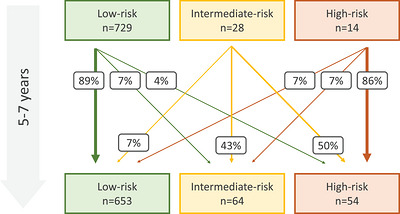
Five‐year changes in risk of Aβ pathology, using plasma p‐tau217 in the Gothenburg H70 Birth Cohort Study. Cutoffs for risk determination according to Palmqvist et al.^5^. Aβ, amyloid‐β.

Over time, there was a slight reduction in individuals classified as low‐risk individuals and a more than three‐fold increase in individuals classified as high‐risk individuals (Figure 2). Most participants remained within the same group between baseline and follow‐up (low‐risk 89% and high‐risk 86%). The intermediate group demonstrates the highest variability with 50% transitioning to the high‐risk group and 7% to the low‐risk group, thus only 43% remained in this group. While only a small proportion of participants from the low‐risk group transitioned to the high‐risk group over 5‐7 years (4%), this small relative contribution still constituted the majority of individuals in the high‐risk group at follow‐up (28 of the 54). This is explained by the large absolute number of individuals in the low‐risk group at baseline (*n* = 729).

### 3.5 Cognitive outcomes at follow‐up depending on baseline risk group

**FIGURE 3:**
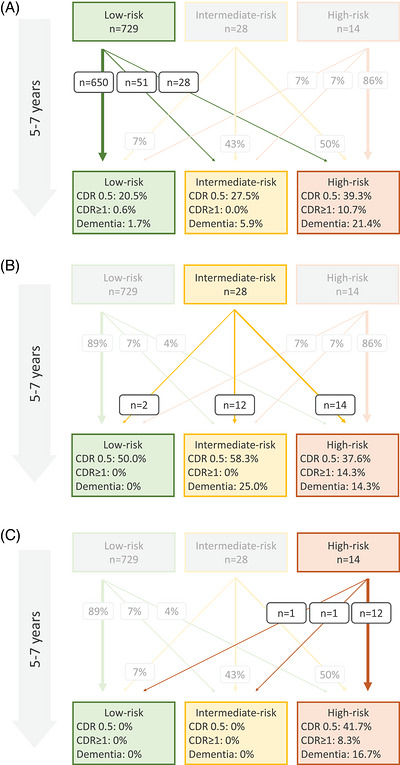
Cognitive outcomes at follow‐up examinations for participants in each plasma p‐tau217 risk group are presented by risk group classification at initial examination. Participants with an initial low‐risk (A), intermediate‐risk (B), and high‐risk (C) are shown.

Participants remaining in the low‐risk group over time had a low proportion with cognitive impairments at follow‐up (Figure 3A). Among individuals transitioning to a higher risk group, there were progressively higher prevalence of MCI (CDR = 0.5) and dementia. Among participants initially classified in the intermediate group, the smaller sample size increased uncertainty, but dementia and CDR ≥ 1.0 was only found among individuals remaining in the intermediate‐risk group or transitioning to the high‐risk group (Figure 3B). In the high‐risk group, the two individuals who transitioned to a lower‐risk group had normal cognitive status defined by CDR (Figure 3C). In contrast, among participants remaining in the high‐risk group, 41.7% had CDR≥0.5 and 16.7% had dementia at follow‐up.

### 3.6 Baseline characteristics of transitioners and non‐transitioners in each risk group

Overall, few differences were found between participants who transitioned between risk groups over time. In the large group with low‐risk individuals, CKD alongside *APOE4* carriership were more common among transitioners (Table 3). Furthermore, these participants also had lower Aβ42 and 42/40‐ratio in CSF. Transitioners in the intermediate group could transition both up (14 of the 16 transitioners) and down (two of the 16 transitioners). All participants with CDR > 0, diabetes, CKD and *APOE* e4 carriership transitioned to the high‐risk category, and all four participants in the CSF subsample transitioned up to the high‐risk category. No differences were observed between participants in the intermediate‐risk or high‐risk groups, possibly due to insufficient sample sizes.

**TABLE 3:** Characteristics of participants in the Gothenburg H70 who stay in the same risk group (non‐transitioners) at follow‐up, or transition to another risk group at follow‐up.

	Low risk			Intermediate risk			High risk		
Parameter	Non‐transitioners	Transitioners	*p*‐Value	Non‐transitioners	Transitioners	*p*‐Value	Non‐transitioners	Transitioners	*p*‐Value
n (%)	650 (89.2)	79 (10.8)		12 (42.9)	16 (57.1)		12 (85.7)	2 (14.3)	
Age, years	70.5 (0.3)	70.5 (0.2)	0.592	70.5 (0.3)	70.5 (0.2)	0.881	70.7 (0.4)	70.4 (0.2)	0.297
Sex, female	343 (52.8)	43 (54.4)	0.780	5 (41.7)	7 (43.8)	0.912	3 (25)	2 (100)	0.110^a^
MMSE, score	29.1 (1.3)	29 (1.1)	0.508	28.8 (1.4)	28.9 (1.7)	0.834	29.2 (1.4)	30 (0)	0.433
CDR = 0	542 (83.6)	65 (82.3)	0.804	9 (75)	13 (81.3)	1.000^a^	9 (75)	2 (100)	1.000^a^
CDR = 0.5	103 (15.9)	13 (16.5)	0.889	3 (25)	3 (18.8)	1.000^a^	3 (25)	0 (0)	1.000^a^
CDR = 1‐3	3 (0.5)	1 (1.3)	0.369^a^	0 (0)	0 (0)	–	0 (0)	0 (0)	–
BMI, kg/m2	25.8 (4.2)	25.3 (4.2)	0.299	25 (7.3)	24.9 (4.6)	0.992	26.4 (3.8)	30.5 (0.9)	0.168
Dementia	4 (0.6)	2 (2.5)	0.131^a^	0 (0)	0 (0)	–	0 (0)	0 (0)	–
Previous stroke	35 (5.4)	5 (6.3)	0.728	2 (16.7)	1 (6.3)	0.560^a^	0 (0)	0 (0)	–
Diabetes	54 (8.3)	10 (12.7)	0.200	1 (8.3)	3 (18.8)	0.613^a^	1 (8.3)	1 (50)	0.275^a^
CKD	46 (7.1)	13 (16.5)	**0.004**	0 (0)	3 (18.8)	0.238^a^	1 (8.3)	0 (0)	1.000^a^
*APOE* ε*4* carrier	189 (29.1)	33 (41.8)	**0.021**	10 (83.3)	7 (43.8)	0.054^a^	5 (41.7)	0 (0)	0.505^a^

CSF substudy, n = 238	*n* = 226			*n* = 7			*n* = 5		
n (%)	211 (93.4)	15 (6.6)		3 (42.9)	4 (57.1)		3 (60)	2 (40)	
Aβ42, pg/mL	566.4 (176.5)	425.1 (197)	**0.003**	497 (325.1)	327.3 (18.2)	0.330	385.3 (348.7)	632.5 (137.9)	0.427
Aβ42/40	0.9 (0.2)	0.7 (0.2)	**<0.001**	0.6 (0.3)	0.5 (0.1)	0.588	0.7 (0.3)	1 (0.1)	0.275
Tau, pg/mL	310.8 (113.9)	360.2 (160.1)	0.116	633.3 (224.9)	442.8 (96.3)	0.181	345.7 (78.1)	327 (46.7)	0.787
p‐tau181, pg/mL	47.1 (15)	52.8 (19.8)	0.163	82 (32)	64.5 (14.8)	0.371	50.3 (2.3)	76.5 (43.1)	0.334
NfL, pg/mL	864.3 (749.9)	1129.1 (1376.3)	0.219	811.7 (267.5)	798.8 (221.4)	0.947	811.3 (405.6)	6385.5 (8381.3)	0.297
Q_Alb_	6.5 (2.4)	6.8 (2.7)	0.607	6.5 (1.8)	5.8 (1.7)	0.616	6 (0.9)	5.8 (0.1)	0.704
Aβ pathology	92 (43.6)	9 (60)	0.217	2 (66.7)	4 (100)	0.429^a^	2 (66.7)	0 (0)	0.400^a^

*Note*: Continous variables were compared with students t‐test and categorical variables with chi‐square test or Fishers exact test

^a^, if low frequency of events. Continuous variables are presented as mean (SD) and categorical variables as n (%).

Abbreviation: Aβ, amyloid‐β; APOE, apolipoprotein E; BMI, body mass index; CDR, Clinical Dementia Rating; CKD, chronic kidney disease; CSF, cerebrospinal fluid; MMSE, Mini‐Mental State Examination; NfL, neurofilament light chain; Q_Alb_, albumin quotient.

### 3.7 The influence of confidence intervals for rate of transition to another risk group over time

The cutoffs used in this study were presented with confidence intervals in the initial publication.^5^ To assess if individuals who transitioned were overrepresented in the uncertain region of the cutoff, we evaluated the proportion of transitioners and non‐transitioners within and outside the uncertain region of the cutoffs (Figure S1).

Participants who did not transition from the low‐risk group were almost all below the uncertain region of the lower cutoff (96.9%). Among individuals transitioning from the low‐risk group, more than one‐in‐three had plasma p‐tau217 measured in the uncertain region. A similar pattern was seen among non‐transitioners in the high‐risk group. Two‐thirds of non‐transitioners were above the uncertain range of the cutoff, compared to 50% who transitioned to a lower risk group. Among intermediate‐risk individuals, there was no clear pattern (Figure S1). In fact, most participants in both the non‐transitioner group and the transitioner group were found in the uncertain region of the cutoffs. Taken together, there was no clear evidence that transitioners were individuals with measurements close to the cutoffs.

## 4 DISCUSSION

We examined the prevalence of high‐risk plasma p‐tau217 levels in a community‐based sample of 1157 70‐year olds and determined the real‐world longitudinal dynamics of plasma p‐tau217 in 771 of these individuals over 5–7 years. The prevalence of individuals at high risk of Aβ pathology, defined by plasma p‐tau217, increases substantially over this time period, alongside clinical and biochemical markers of neurodegeneration and AD. We also found that a few individuals without clinical signs of dementia normalised elevated levels of p‐tau217.

It is often stated that the prevalence of dementia doubles every 5‐years from the age of 70^12^. Here, we found that the proportion of individuals at high‐risk of Aβ pathology defined by plasma p‐tau217 also doubled in the same time span. We have previously reported that the prevalence of Aβ pathology defined by pathological CSF Aβ42 concentrations is 22.8% among community‐dwelling 70‐year‐olds.^22^ A population‐based study in the UK reported that 19% of 70‐year olds are amyloid‐PET positive.^24^ This suggests that plasma p‐tau217 identifies a narrower subgroup of cognitively healthy individuals at risk of future dementia. The cutoffs for plasma p‐tau217 have been established in independent cohorts of individuals with cognitive symptoms from memory clinics and primary care with excellent accuracy ^5^. A recent community‐based study reported that plasma p‐tau217 identifies a larger proportion of individuals with amyloid positivity in individuals with cognitive impairment than cognitively unimpaired individuals and that the plasma p‐tau217 performance increased with age.^25^ As the absolute majority of participants here did not have any cognitive impairment and were relatively young at the initial examination, this may explain the discrepancy between high‐risk plasma p‐tau217 and Aβ pathology defined by pathological CSF Aβ42. Several studies have previously demonstrated that plasma p‐tau217 identifies Aβ pathology in both clinical and community‐based settings,^5^, ^11^ but information on the difference in diagnostic performance between CSF Aβ‐42 and plasma p‐tau217 on predicting amyloid PET is limited. We also find that individuals with high‐risk levels of plasma p‐tau217 have worse Aβ biomarkers and cognitive performance at follow‐up, suggesting that the marker is useful to identify community‐dwelling individuals at high risk of developing AD. Similar observations have previously been reported in memory clinic patients, suggesting that plasma p‐tau217 is useful over the full AD continuum.^7^ The proportion of men and women with high‐risk plasma p‐tau217 were similar, without indication that sex had any confounding influence.

At baseline, there were no differences in clinical markers between the different risk groups. This aligns with current knowledge that biochemical manifestations are present before clinical symptoms appear. Our observations suggest that plasma p‐tau217 can identify early pathological variations in community‐dwelling 70‐year‐olds, even if the prevalence of cognitive symptoms is low. This observation extends previous reports that plasma p‐tau217 predicts amyloid deposition in populations at high risk of developing AD^8^. Our observation aligns with other community‐based reports,^11^, ^26^ demonstrating the usefulness of plasma p‐tau217 as a marker of AD pathology in a general population with a low proportion of cognitive impairment.^27^

Interestingly, risk stratification at the age around 75 years resulted in significant group‐differences in both CDR‐rating, dementia prevalence, and MMSE score. Differences may manifest at this higher age as the prevalence of cognitive impairment is also higher. This shows that plasma p‐tau217 can be used both in asymptomatic 70‐year‐olds to screen for early biochemical changes, and older patients with manifest cognitive decline. The performance of plasma p‐tau217 as a biomarker is manifested as the proportion of individuals with dementia in the high‐risk group was almost 10‐times higher than in the low‐risk group at follow‐up (2% vs. 19%).

Almost 90% of participants from the low‐risk group remained there at follow‐up. The high proportion suggests that the test has long term reliability for a large majority of individuals. It should be noted that even though only a small proportion transitioned from low‐ to high‐risk over 5–7 years, these individuals constituted a substantial proportion of all individuals at high‐risk at follow‐up. This was due to the large absolute number of low‐risk individuals at baseline. Individuals transitioning from low‐ to high‐risk may be individuals with a more aggressive pathology. Combining complementary strategies to also identify rapid transitioning individuals early on will be an important task going forward. We found that the risk of transitioning to a higher‐risk category was lower if you had a plasma p‐tau217 value outside the uncertain area of the cutoff. This should be considered when these cutoffs are used in a clinical setting. We also found that CKD and *APOE4* carriership were risk factors for transition over the follow‐up time. This may indicate that a combination of these factors could yield a higher performing diagnostic instrument for rapid transitioners.^28^, ^29^ We recently demonstrated that plasma‐based apoE proteotyping can be done with relatively high precision.^30^ It may thus be possible to determine plasma p‐tau217, CKD, and *APOE4* carriership with a single plasma test. Future studies could evaluate if such strategy could give a higher precision to determine cognitive decline.

Two individuals in the high‐risk category transitioned down to a lower category at follow‐up. While both these individuals were *APOE4* non‐carriers and had CDR = 0 at follow‐up, they did present with relatively high levels of both p‐tau181 and neurofilament light in CSF and, thus, still indicate underlying neurodegenerative pathology. Larger samples of high‐risk individuals would be necessary to better understand why some individuals can transition down in plasma p‐tau217 defined risk category.

The intermediate‐risk subgroup presented the lowest levels of Aβ42/40 and the highest levels of tau and ptau181 in the CSF subsample. This was unexpected and would have been expected in the high‐risk group with highest rate of cognitive decline. In contrast, the neurofilament light chain concentrations demonstrated progressive increase over subgroups, aligning with the high sensitivity as a marker of neurodegeneration. The observations among amyloid and tau markers may partly be explained by a limited statistical power with small groups of individuals in the intermediate and high‐risk subsamples with CSF. However, it should be noted that the prevalence of *APOE4* carriership was also highest in the intermediate‐risk group at both baseline and follow‐up, where statistical power was higher. This could indicate an underlying biological difference between the groups. While not statistically significant, the four participants with CSF in the intermediate group, who transitioned to a higher risk‐category at follow‐up also presented levels of tau and p‐tau181 in in the higher range in relation to those remaining in the intermediate group. Possible underlying processes behind these observations cannot be ruled out and should be examined in designated studies.

We also found that the change in plasma p‐tau217 was significantly higher among individuals progressing in CDR from both CDR = 0 and CDR = 0.5 at baseline. This is likely a manifestation of ongoing pathophysiological processes. We did not find any meaningful diagnostic value for using the change of plasma p‐tau217 over time as a predictor of cognitive decline in this community‐based sample, although different results may be found in clinical samples with a higher prevalence of neurodegenerative disease.

There are some limitations to consider in this study. We mainly examined participants with a European ancestry. The prevalence of cognitive disease was similar to what has previously been reported in non‐selected individuals from the United States, but low in relation to what is found on studies in memory clinics, who may thus find different results.^31^ The low frequency of individuals classified as intermediate‐risk and high‐risk limits the power for deeper analysis of these sub‐groups. However, this cohort is representative of a general population of 70‐year‐olds in Sweden, thus providing useful information on plasma p‐tau217 in a real‐world setting.^32^ There was a 33% loss to follow‐up over the study time with a higher proportion of participants lost in the high‐risk category. However, there was no difference in cognitive variables between the risk categories among those not returning for re‐examination, nor for the reason given to not return. The potential influence of these lost participants should be considered when interpreting this study, as participants with several different diseases are more likely to not be re‐examined. Regardless, we still find that high plasma p‐tau217 is associated with cognitive decline although the effect size may be underestimated.

In conclusion, repeated measurements of plasma p‐tau217 in community‐dwelling 70‐year‐olds showed that it is a reliable biomarker with high stability over a 5–7 year follow up period. Individuals who transitioned to a higher risk‐category over time also had a higher prevalence of cognitive disability at follow‐up, demonstrating increased risk for future cognitive decline among community‐dwelling 70‐year‐olds. However, the risk of developing dementia was less than 25% even in the highest risk groups over a 5–7 year follow‐up time.

## CONFLICT OF INTEREST STATEMENT

Anna Dittrich, Burak Arslan, Lina Rydén, Hlin Kvartsberg, Kaj Blennow, Ingmar Skoog, and Johan Gobom report no conflicts of interests. T.S. has served at a scientific advisory board for Quantify Research unrelated to present study content. Author disclosures are available in the Supporting Information.

HZ has served at scientific advisory boards and/or as a consultant for Abbvie, Acumen, Alector, Alzinova, ALZpath, Amylyx, Annexon, Apellis, Artery Therapeutics, AZTherapies, Cognito Therapeutics, CogRx, Denali, Eisai, Enigma, LabCorp, Merck Sharp & Dohme, Merry Life, Nervgen, Novo Nordisk, Optoceutics, Passage Bio, Pinteon Therapeutics, Prothena, Quanterix, Red Abbey Labs, reMYND, Roche, Samumed, ScandiBio Therapeutics AB, Siemens Healthineers, Triplet Therapeutics, and Wave, has given lectures sponsored by Alzecure, BioArctic, Biogen, Cellectricon, Fujirebio, LabCorp, Lilly, Novo Nordisk, Oy Medix Biochemica AB, Roche, and WebMD, is a co‐founder of Brain Biomarker Solutions in Gothenburg AB (BBS), which is a part of the GU Ventures Incubator Program, and is a shareholder of CERimmune Therapeutics (outside submitted work). S.K. has served at scientific advisory boards, speaker and / or as consultant for Roche, Eli Lilly, Geras Solutions, Optoceutics, Biogen, Eisai, Merry Life, Triolab, Novo Nordisk and Bioarctic, unrelated to present study content. Author disclosures are available in the Supporting Information.

## CONSENT STATEMENT

This study was conducted according to the Helsinki Declaration approved by the Regional Ethical Review Board in Gothenburg. All the participants and/or their close relatives gave written consent before any study related procedures.

## Supporting information




**Supporting Information**: alz71545‐sup‐0001‐SuppMat.docx

**Supporting Information**: alz71545‐sup‐0002‐SuppMat.pdf
