# Supplementary material for: Prevalence of high‐risk plasma p‐tau217 levels and 5‐year transition of risk status in 70‐year‐olds
Source: Alzheimers Dement. 2026 Jun 7;22(6):e71545. doi: 10.1002/alz.71545 (PMC13243198; doi:10.1002/alz.71545)
Supplement: Supplementary file 1 — Supporting Information: alz71545‐sup‐0001‐SuppMat.docx [file ALZ-22-e71545-s002.docx]

**Supplementary material**

**Supplementary figure 1
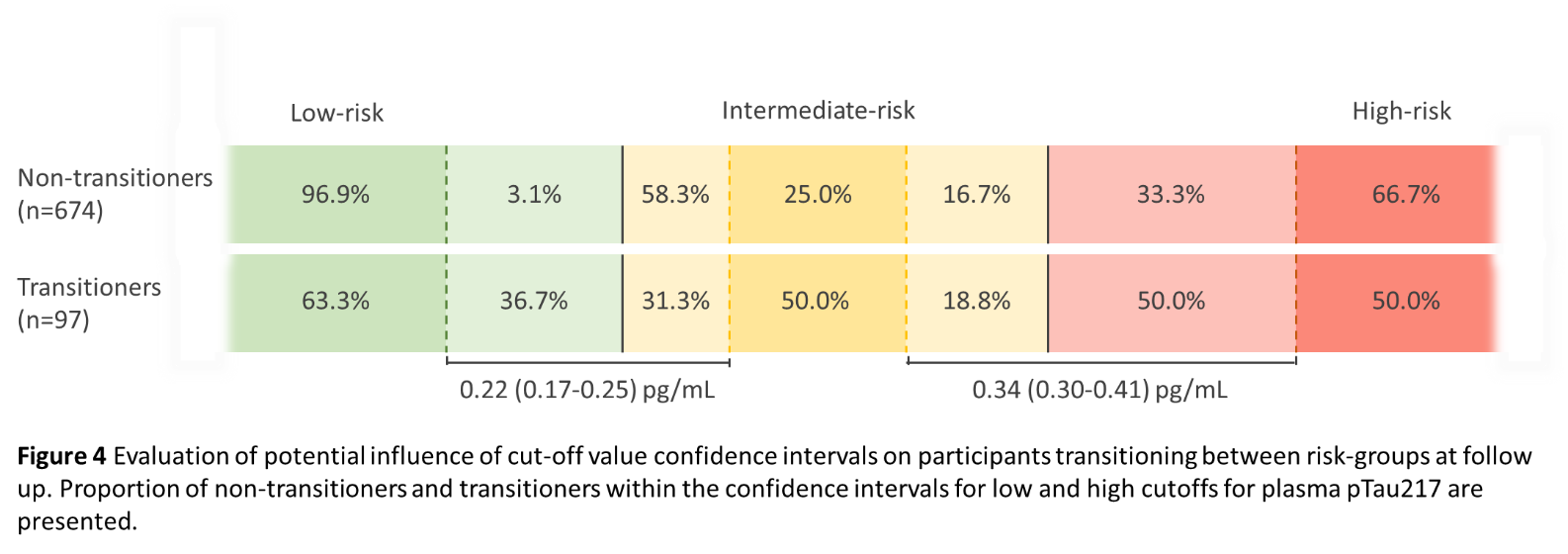
**

**Supplementary figure 1** Evaluation of potential influence of cut-off value confidence intervals on participants transitioning between risk groups at follow up. Proportion of non-transitioners and transitioners within the confidence intervals for low and high cut-offs for plasma p-tau217 are presented.

| **Supplementary table 1** Baseline characteristics of participants lost to follow-up for the different risk groups. | | | | |
| --- | --- | --- | --- | --- |
|  | Low-risk | Intermediate-risk | High-risk | *p*-value |
| N | 338 | 20 | 28 |  |
| Declined follow-up | 198 (58.6) | 14 (70) | 15 (53.6) | 0.510 |
| No plasma p-tau217 at follow-up | 39 (11.5) | 4 (20) | 5 (17.9) | 0.299 |
| Deceased at follow-up | 26 (7.7) | 0 (0) | 0 (0) | 0.176 |
| Uncontactable at follow-up | 28 (8.3) | 1 (5) | 2 (7.1) | 1.000 |
| Other reason for loss to follow-up | 47 (13.9) | 1 (5) | 6 (21.4) | 0.299 |
| Age, years | 70.6 (0.3) | 70.5 (0.2) | 70.6 (0.2) | 0.149 |
| Sex, female | 179 (53) | 11 (55) | 19 (67.9) | 0.314 |
| MMSE, score | 28.6 (2.7) | 29.1 (1.3) | 27.6 (2.8) | 0.149 |
| CDR=0 | 244 (72.2) | 14 (70) | 15 (53.6) | 0.114 |
| CDR=0.5 | 78 (23.1) | 6 (30) | 11 (39.3) | 0.136 |
| CDR=1-3 | 10 (3) | 0 (0) | 1 (3.6) | 0.773 |
| BMI, kg/m^2^ | 26.8 (5) | 25.1 (3.3) | 26.2 (5.7) | 0.315 |
| Dementia diagnose | 16 (4.8) | 0 (0) | 4 (14.3) | 0.070 |
| Previous stroke | 26 (7.7) | 2 (10) | 1 (3.6) | 0.663 |
| Diabetes | 60 (17.8) | 3 (15) | 4 (14.3) | 0.557 |
| CKD | 40 (11.9) | 6 (30) | 8 (28.6)* | **0.005** |
| *APOE ε4*-carrier | 105 (31.1) | 10 (50) | 17 (60.7)* | **0.002** |
| Abbreviations: BMI - Body Mass Index, CDR - Clinical Dementia Rating, CKD - Chronic Kidney Disease, CSF - Cerebrospinal fluid, MMSE - Mini Mental State Examination, Q_alb_ - Albumin quotient. Continuous variables are presented as mean (SD) and categorical variables as n (%).  Continuous variables were compared with one-way ANOVA and categorical variables with Chi^2^-test or Fishers exact test^a^, if low frequency of events.  * Difference compared to low-risk group, with Bonferroni-adjusted z-test for categorical variables. | | | | |

| **Supplementary table 2** Longitudinal characteristics of participants stratified according to cut-offs for plasma p-tau217 at the baseline examination. | | | | | | | | | | | | |
| --- | --- | --- | --- | --- | --- | --- | --- | --- | --- | --- | --- | --- |
|  | Low-risk at baseline (n=729) | | |  | Intermediate-risk at baseline (n=28) | | |  | High-risk at baseline (n=14) | | | |
|  | Baseline | Follow-up | *p*-value |  | Baseline | Follow-up | *p*-value |  | Baseline | Follow-up | *p*-value | |
| Age, years | 70.5 (0.3) | 76.3 (0.6) | **<0.001** |  | 70.5 (0.3) | 76.5 (0.8) | **<0.001** |  | 70.7 (0.4) | 76.2 (0.6) | **<0.001** | |
| Sex, female | 386 (53) | 386 (53) | - |  | 12 (43) | 12 (43) | - |  | 5 (36) | 5 (36) | - | |
| MMSE, score | 29.1 (1.3) | 28.7 (1.6) | **<0.001** |  | 28.8 (1.5) | 27.0 (3.1) | **0.005** |  | 29.3 (1.3) | 28.4 (2.3) | **0.031** | |
| CDR=0 | 607 (83) | 564 (77) | **0.005** |  | 22 (79) | 13 (46) | **0.013** |  | 11 (79) | 8 (57) | 0.420^a^ | |
| CDR=0.5 | 116 (16) | 158 (22) | **0.005** |  | 6 (21) | 13 (46) | **0.048** |  | 3 (21) | 5 (36) | 0.678^a^ | |
| CDR=1-3 | 4 (1) | 7 (1) | 0.547^a^ |  | 0 (0) | 2 (7) | 0.491^a^ |  | 0 (0) | 1 (7) | 1.000^a^ | |
| BMI, kg/m2 | 25.8 (4.2) | 25.7 (4.3) | 0.140 |  | 24.9 (5.8) | 24.6 (5.8) | 0.402 |  | 27 (3.9) | 25.6 (3.6) | 0.065 | |
| Dementia | 6 (1) | 20 (3) | **0.006** |  | 0 (0) | 5 (18) | 0.052^a^ |  | 0 (0) | 2 (14) | 0.482^a^ | |
| Previous stroke | 40 (5) | 53 (7) | 0.164 |  | 3 (11) | 3 (11) | 1.000^a^ |  | 0 (0) | 1 (7) | 1.000^a^ | |
| Diabetes | 64 (9) | 97 (13) | **0.006** |  | 4 (14) | 4 (14) | 1.000^a^ |  | 2 (14) | 3 (21) | 1.000^a^ | |
| CKD | 59 (8) | 104 (14) | **<0.001** |  | 3 (11) | 4 (14) | 1.000^a^ |  | 1 (7) | 1 (7) | 1.000^a^ | |
| *APOE* ε*4* carrier | 222 (30) | - | - |  | 17 (61) | - | - |  | 5 (36) | - | - | |
| Abbreviations: BMI - Body Mass Index, CDR - Clinical Dementia Rating, CKD - Chronic Kidney Disease, MMSE - Mini Mental State Examination. Continuous variables are presented as mean (SD) and categorical variables as n (%).  Continuous variables were compared with paired t-test and categorical variables with Chi^2^-test or Fishers exact test^a^, if low frequency of events. | | | | | | | | | | | |  |
